# Frame localisation optical projection tomography

**DOI:** 10.1038/s41598-021-83454-z

**Published:** 2021-02-25

**Authors:** Craig T. Russell, Pedro P. Vallejo Ramirez, Eric Rees

**Affiliations:** 1 Department of Chemical Engineering and Biotechnology, Cambridge University, Cambridge, UK; 2 European Bioinformatics Institute, Wellcome Genome Campus, Cambridge, CB10 1SD UK

**Keywords:** Imaging and sensing, Microscopy

## Abstract

We present a tomographic reconstruction algorithm (flOPT), which is applied to Optical Projection Tomography (OPT) images, that is robust to mechanical jitter and systematic angular and spatial drift. OPT relies on precise mechanical rotation and is less mechanically stable than large-scale computer tomography (CT) scanning systems, leading to reconstruction artefacts. The algorithm uses multiple (5+) tracked fiducial beads to recover the sample pose and the image rays are then back-projected at each orientation. The quality of the image reconstruction using the proposed algorithm shows an improvement when compared to the Radon transform. Moreover, when adding a systematic spatial and angular mechanical drift, the reconstruction shows a significant improvement over the Radon transform.

Sharpe et al. proposed OPT^1^ using visible light to image transparent or translucent mesoscopic samples, with micrometer resolution. OPT addresses the scale gap between photographic techniques (for samples typically larger than 10 mm), and light microscopy techniques (samples smaller than 1 mm) to image biological samples in the 1 mm to 10 mm range.

OPT is based on computerised tomography techniques^2^ in which a set of projections of a specimen are acquired as the specimen travels through a full rotation, shown in Fig. 1b. Typically, a Radon transform is then used to transform this set of images into a 3D image stack in Cartesian coordinates (*X*, *Y*, *Z*). The Radon transform relies heavily on the assumption of circular motion with constant angular steps about a vertical axis. Prior to the Radon transform, an attempt is made to find the centre of rotation (CORs) and correct the image shift^3–5^; this step is both computationally expensive, error prone and incomplete with regards to all available degrees of freedom. This work presents an improved general reconstruction algorithm that is robust to spatial and angular mechanical drifts during acquisitions, as well as to inconsistent angular steps. The proposed algorithm triangulates points between image pairs to extract camera pose using the theoretical framework used in stereoscopic imaging.

**Figure 1:**
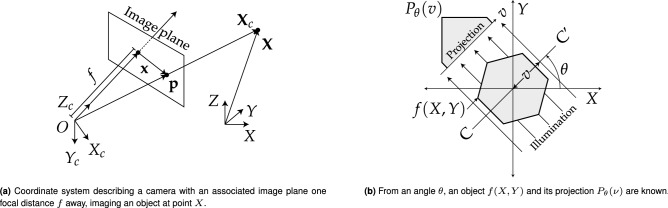
$\mathbf{X}_\mathbf{c} = (X_c,Y_c,Z_c)$ is the camera-centered coordinate point in 3D space. $\mathbf{X} = (X,Y,Z)$ is the world coordinate point in 3D space. $\mathbf{p} = (x,y,f)$ is the ray vector to point of image plane. $\mathbf{x} = (x,y)$ is the image plane coordinates. $\mathbf{w} = (u,v)$ are the pixel coordinates (not shown) corresponding to the point **x**. The optical axis travels along the $Z_c$ axis through the image plane.

## Stereoscopic imaging

**Figure 2:**
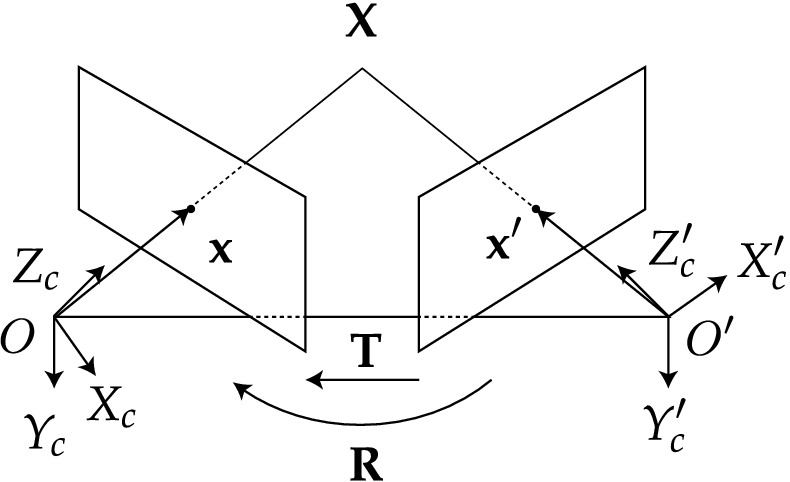
Epi-polar geometry described for two adjacent views (or cameras of a scene). Coordinates as expressed in Fig. 1a with prime notation ($^{\prime}$) denoting the additional right camera view. Transforming from right to left camera-centered coordinates ($\mathbf{X}^{\prime}_\mathbf{c}$ to $\mathbf{X}_\mathbf{c}$) requires a rotation (**R**) and a translation (**T**).

When the features or fiducial markers in one view are uniquely identifiable, the stereoscopic imaging of scenes allows for the triangulation of individual features in three dimensional space (known as world points), see Figs. 1 and 2 for the coordinate system which describes this geometry. Triangulation requires that each feature is detected in both images of a stereo imaging system and for these detections to be correctly associated with one another. This is known as the correspondence problem. Various methods exist to ensure that features are detected from image data and accurately associated between two cameras or views^6^ and the properties of scale-independent features and their surrounding pixel environment in one image can thus be matched to a similar feature in a second image.

Coordinates in two adjacent views with a common epi-pole (the vector connecting the *O* and $O^{\prime}$, see Fig. 2) are related by the essential matrix (**E**) for uncalibrated cameras and the fundamental matrix (**F**) for calibrated cameras. Their properties are described by:

1 $$\begin{aligned}&\mathbf{p}^{{\prime}T} \mathbf{Ep} = 0 \end{aligned}$$

2 $$\begin{aligned}&\mathbf{E} = \mathbf{K}^{{\prime}T} \mathbf{F} \mathbf{K} \end{aligned}$$

where $\mathbf{K}$ is a matrix that converts image plane coordinates to camera pixel coordinates and where **p** refers to a point in the image plane.

## The proposed algorithm (flOPT)

The motion of a rotating sample, as in an OPT acquisition, with a transformation matrix ($[\mathbf{R} \,|\, \mathbf{T}]$) in view of a fixed camera is analogous to the motion of a camera around the scene with the inverse transformation matrix. During an ideal OPT acquisition, a marker will appear to follow an elliptical path in the *xy* image plane. For the volume reconstruction procedure, there is a fitting step to recover the path of the fiducial marker, which is used to correct the sinogram before applying the inverse Radon transform. This type of reconstruction not only ignores any mechanical jitter of the sample, but also any affine, systematic, mechanical drift (in $X,Y,Z,\theta ,\phi ,\psi$). This can be rectified by recovering the complete non-scaling transformation for every projection. Now, using two adjacent images of a scene (separated by some rotation and translation) world points in 3D space may be triangulated within the scene given the rotational and translational matrices of the respective camera views.

Once a sufficient amount of fiducial markers are reliably tracked from the first to the second image, either one of the fundamental or essential matrices can be computed. Using the factorisation of one of these matrices, between each adjacent view of a rotating scene, the translation and rotational matrices can be recovered.
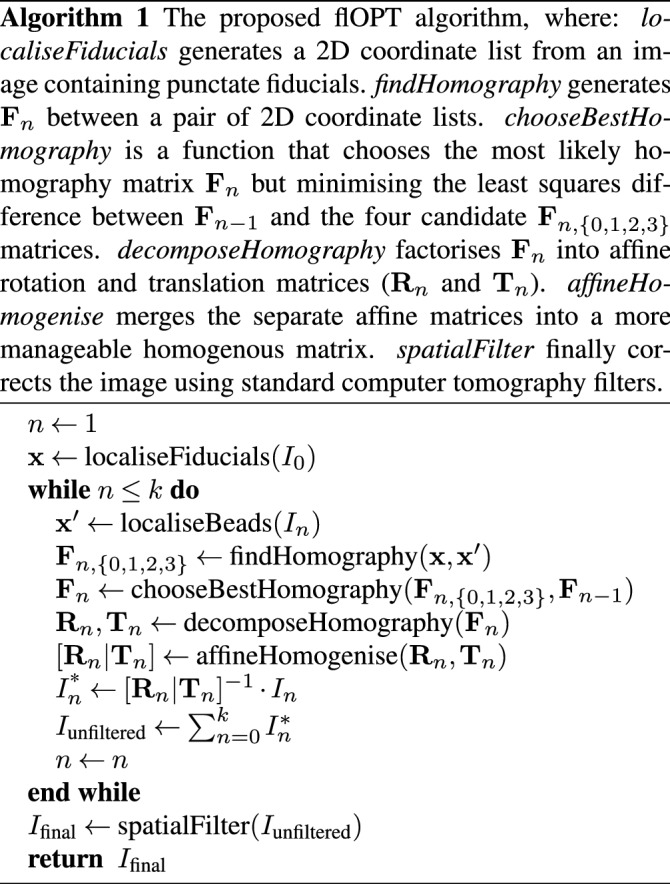


**Figure 3:**
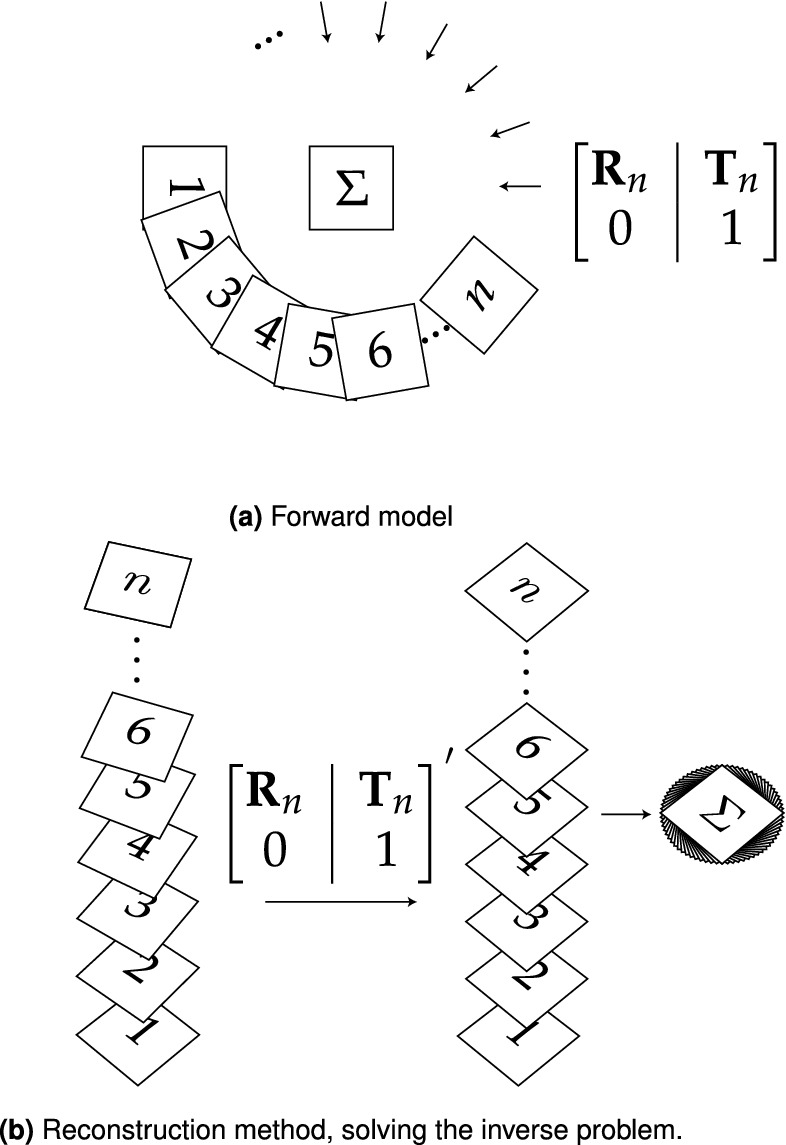
The simulation of OPT data incorporating rotational and translational offsets, and the proposed reconstruction algorithm. (**a**) The *n* projections of the object ($\Sigma$), at rotation ($\mathbf{R}_1$ to $\mathbf{R}_n$) and translation ($\mathbf{T}_1$ to $\mathbf{T}_n$), produces *n* frames of image data. During the OPT measurement, *n* projections of the object $\Sigma$ are observed with rotations $\mathbf{R}_1$ to $\mathbf{R}_n$ and corresponding translations $\mathbf{T}_1$ to $\mathbf{T}_n$ where the translations account for imperfect alignment. (**b**) In the reconstruction algorithm, the rotational and translational matrices are recovered ($\mathbf{R}_1^{\prime}$ to $\mathbf{R}_n^{\prime}$ and $\mathbf{T}_1^{\prime}$ to $\mathbf{T}_n^{\prime}$) from triangulation of the fiducial markers. These transformation matrices are then used to obtain a contribution to the volumetric reconstruction from each observed frame and the summated reconstruction is assembled from the *n* frames. The now realigned back projections are summed to produce an unfiltered back projection. The transformation matrices are shown in augmented form using homogenous coordinates.

To reconstruct the image, we compute **F** for the current image and the first image using 5 or more fiducial markers; having additional beads helps to remove ambiguity and increase confidence in **F**. Once **F** is calculated, it is decomposed into $\mathbf{R}_n$ and $\mathbf{T}_n$ between each view *n* and $n+1$. The image at view $n+1$ is then back projected along the virtual optical axis within a virtual volume where the sample will be reconstructed. The size of this back projection and virtual volume is chosen to be suitably large, preventing the loss of important data. The recovered transformation matrices are then matrix inverted and applied to the back projection of the image to realign the rays in the volume to their respective source positions as shown in Fig. 3.

In both cases, a decomposed **F** matrix will produce four possible transformation pairs (**R**, **T**; **R**, − **T**; − **R**, **T**; − **R**, − **T**). Once the transformation matrix between the current view (*n*) and the first view is calculated, the proceeding transformation matrices are then easily chosen by similarity to the previously collected matrix and general direction of motion. An example of this type of selection would be:

3 $$\begin{aligned} \min _{I(n)}\left[ I(n) = \left( [\mathbf{R}_n \,|\, \mathbf{T}_n] -[\mathbf{R}_{n-1} \,|\, \mathbf{T}_{n-1}]\right) ^2\right] \end{aligned}$$

To find the correct matrix between the $n=0$ and $n=1$ orientations, each of the four matrices are compared to an ideal matrix which is composed using *a priori* knowledge of the likely angle of rotation of the system’s imaging properties.

## Verification of the proposed algorithm

**Figure 4:**
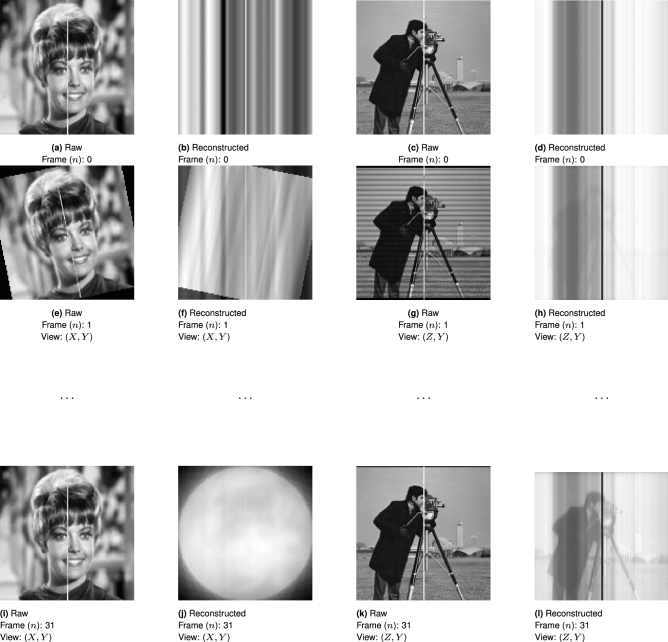
A 3D test-volume of two orthogonal and different testcard images, was used to verify the reconstructive capabilities of the proposed algorithm. The projected image data (**b**,**f**,**j**) and (**d**,**h**,**l**), are projected from (**a**,**e**,**i**) and (**c**,**g**,**k**) respectively and were used to iteratively generate reconstructions where the $n\text {th}$ reconstruction incorporates all the information from observation 0 to *n*. The results are unfiltered for clarity of demonstrating the iterative reconstruction, which is applied in Fig. 6d.

To verify the validity and quality of the proposed reconstruction algorithm, the image of Zelda, superposed with an orthogonal image of Cameraman, is used as a testcard volume. Virtual fiducial beads are dispersed in the volume to track the rotation and translation of the image. The reference image is then rotated through 128 angles over $2\pi$ radians and projected along the *Y* axis, then an image slice in (*X*, *Y*) is taken to create a single line projection, shown three dimensionally in Fig. 4. This is repeated for each angle, with each line projection stacked to create a sinogram.

**Figure 5:**
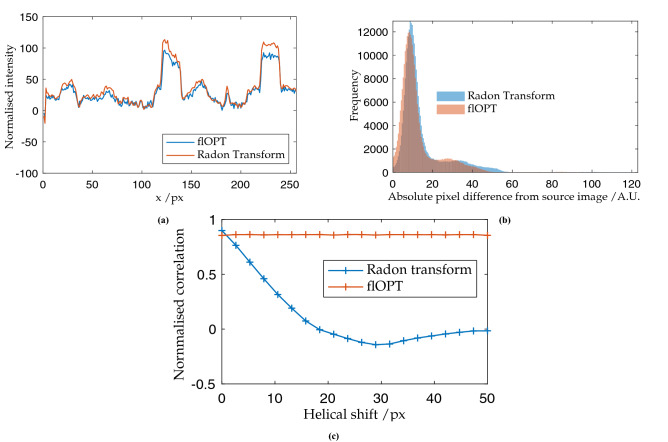
(**a**) Line profile comparison of the reconstruction of a reference image computationally rotated, projected and reconstructed using the standard Radon transform and the new proposed algorithm. (**b**) Histogram of pixel values compared between reconstructions using the new proposed flOPT algorithm and the Radon transform. The shift of the histogram towards overall lower deviance from the source image suggests the flOPT algorithm outperforms the Radon transform. (**c**) Comparison of standard and proposed OPT reconstruction algorithms for acquisitions with drift. 2D image correlation of the ground truth and the reconstruction shows that the proposed flOPT algorithm does not degrade with systematic drift, whereas a reconstruction using the standard Radon transform is severely degraded.

**Figure 6:**
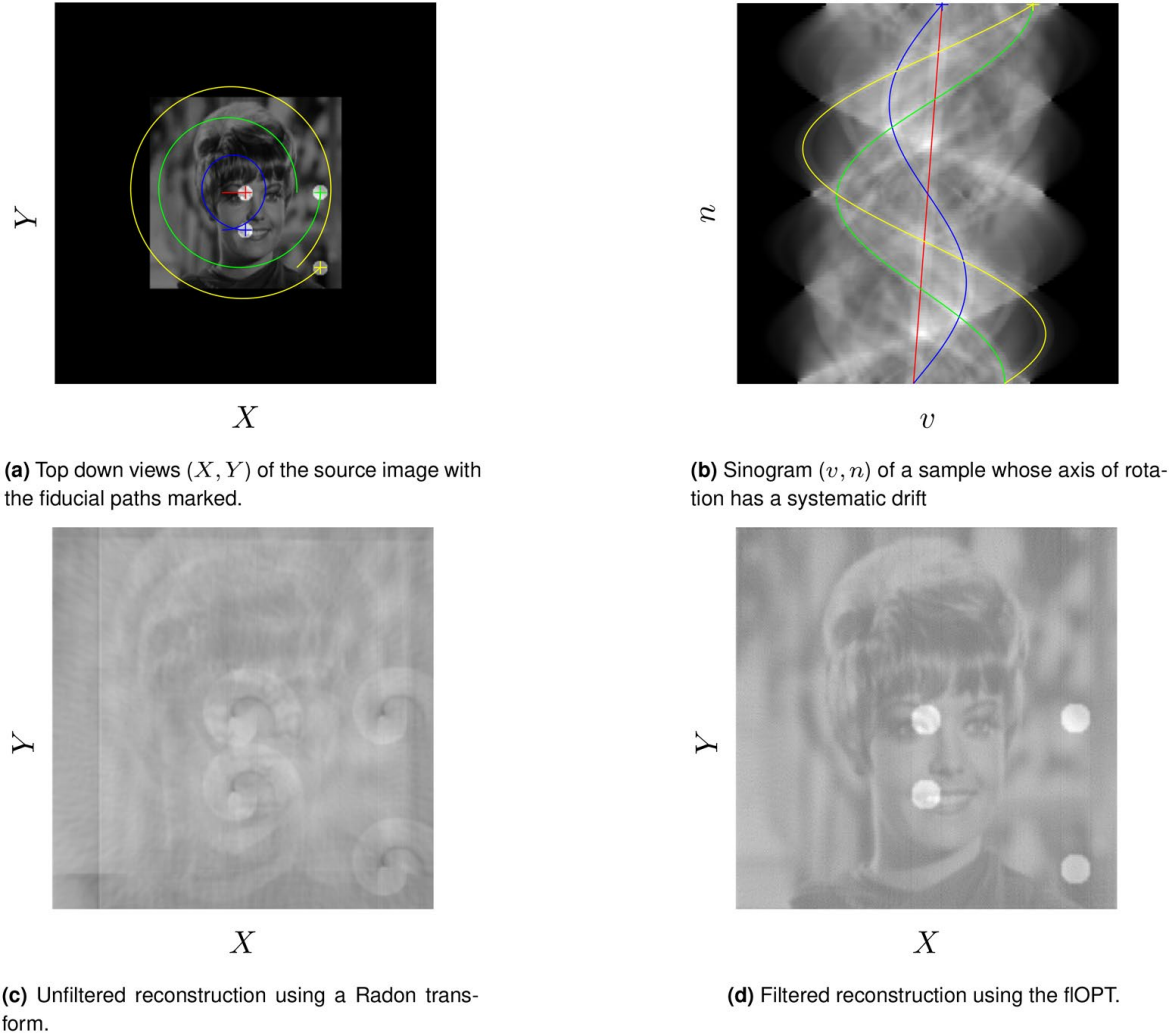
Comparison of the two reconstructions under sample imaging with a systematic drift, in 3D though represented here in 2D. (**a**) Shows the path of four fiducial markers under helical drift; (**b**) shows the sinogram of this motion; with (**c**) showing the result of the Radon; transform on tomographic dataset that contains this corruption whilst (**b**) shows the result of the reconstruction using the flOPT algorithm^7^.

In the standard approach for OPT reconstruction, the sinogram undergoes the inverse Radon transform, as shown in Fig. 4j, followed by post-filtering. This step is substituted for the proposed algorithm; in Fig. 5a the two techniques are compared for ideal conditions of smooth, predictable rotation. The proposed algorithm produces a faithful reconstruction on the original image, as shown in Fig. 6d. Fig. 5b illustrates the strong overlap of the images produced by the new algorithm and the Radon transform when considering the histogram of the absolute pixel-wise difference between the original source image and the respective reconstructions. The proposed algorithm generates lower deviance from the source image than the Radon transform. The mean square errors (MSE, see Eq. (4)) of the new algorithm and the Radon transform are 15.01% and 14.84%, respectively, see Fig. 5b for a histogram of a pixel-wise comparison.

4 $$\begin{aligned} \hbox {MSE}=\frac{1}{n}\sum _{i=1}^n{(Y_i-\hat{Y_i})}^2 \end{aligned}$$

where $\mathbf {Y}$ is the vector of observed values and $\hat{Y_i}$ is mean of the ith value of the predicted values

The more challenging case of a sample drifting systematically along the *X* axis, with a constant velocity, was then considered. This drift produced a helical path of a single fiducial within the sample, see Fig. 6b. In Fig. 6c, the Radon transform fails to produce a recognisable reproduction of the test image with the addition of a slight helicity to the rotation. The proposed algorithm produces an equivalent result to that of a sample rotating without any systematic drift, see Fig. 6c. In Fig. 5c the respective reconstructions from each algorithm were compared, as before, while the helical shift was incremented. See Fig. 6b for a sinogram of a sample (shown in Fig. 6a) wherein a helical shift has been induced. When using correlation as a metric of reproduction quality, the new algorithm fares slightly worse at zero helicity, with 94% correlation compared to the Radon transform at 96%. As expected, the Radon transform rapidly deteriorates once a systematic drift is applied, whereas the new algorithm maintains the quality of the reconstruction, see Fig. 5c.

### Recovery of R and T using matrix decomposition

**Figure 7:**
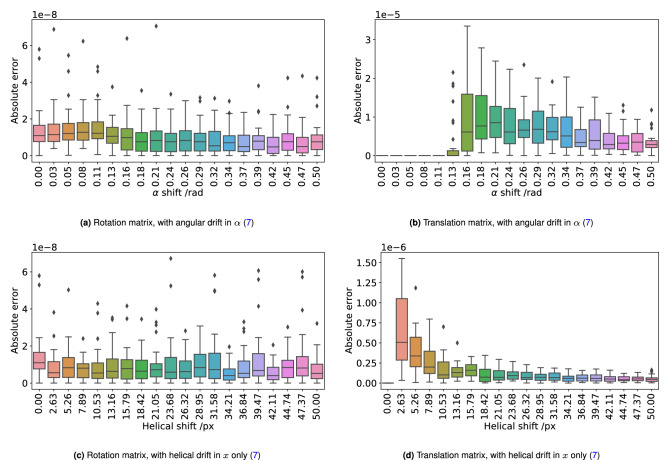
Box plots demonstrating that the rotational and translations matrices can be recovered accurately from fiducial marker positions. Panels (**a**,**b**) introduce an angular drift during rotation, to an observer at the detector this would appear as a tip of the sample towards them, causing precession. Panels (**c**,**d**) introduce a lateral drift in *X* causing a helical path to be drawn out. In all cases, the percentage error introduced by the the addition of undesirable additional movements was on the order of $<2\%.$(note that errors in recovering translation are much larger given a smaller helical shift as the percentage error of the recovery of the translation matrix is broadly constant).

To quantitatively verify that the matrix decomposition technique was valid and robust, the accuracy of the reproduction of **R** and **T** was tested directly. The original **R** and **T** matrices were computed and compared to **R** and **T** generated from matrix decomposition. This absolute difference was computed element-wise in each matrix and then an average for each matrix was taken. Overall, the worst-case scenario produced a percentage error of 2% (see Fig. 7 for full statistics). The accuracy of the calculated **R** and **T** deteriorated when adding in additional degrees of combined movement, but with no correlation between the degree of helicity and the error produced. The translation matrix (**T**) was consistently more accurately reproduced, which is likely due to it having fewer available degrees of freedom.

## Discussion

A new algorithm for reconstructing OPT data has been demonstrated. The new algorithm uses multiple fiducial markers to recover the matrix which describes the rotation and translation of the sample. The quality of the reconstructions shows a slight improvement when compared to the standard Radon transform, with a great effect when a systematic drift is introduced. The accuracy of the decomposition of **F** into **R** and **T** was compared to the ground truth matrices. The element-wise absolute difference $\left( \frac{x-y}{2(x+y)}\right)$ of each matrix was averaged across the matrix for **R** and **T**. In the worst-case scenario, a maximum of 2% average absolute difference was found between ground truth and recovered matrices, suggesting that the technique is robust to various forms of drift in all dimensions and general instability. Such an algorithm could be used to minimise ghosting effects seen in real samples, particularly in samples where slipping is likely to occur, such as in gels or in cheaper OPT systems which tend to be more mechanically unstable and imprecise. In particular the imaging of large mobile gels is set to become more prevalent given the surge of new techniques in Expansion Microscopy^8^, whereby fragile expanded samples embedded in thin lubricious gels.

## Future work

The proposed algorithm relies on triangulation between two view points. However, it is possible to use three separate views to reconstruct a scene, one such approach being quaternion tensors^9^. Working with tensors is more complex, but a future iteration of the algorithm presented here may benefit from using three views to provide a more accurate transformation matrix. Beyond three views, there is currently no mathematical framework for four or more views. If such tools were to be developed, it may be possible to have the algorithm described above be a non-iterative, single-shot reconstruction from pixels to voxels.

Fiducial markers could also be extracted from the image texture alone, circumventing the need for the additional beads embedded in the sample. To find such correspondences, points with similar local texture are found and matched in between each image using standard algorithms such as SIFT^10^ and RANSAC^11^. This was attempted in this work, however, the errors introduced into the transformation matrices make this approach currently unviable; and so by requiring bright punctuate fiducial markers the burden of collecting the fiducial coordinates is shifted to well established curve fitting algorithms that are robust to noise.

## Supplementary Information

Supplementary Information.

## Data Availability

All of the code presented here is FOSS using OpenCV and Python for our simulation, and can be found on GitHub^7^.
