# Supplementary material for: Frame localisation optical projection tomography
Source: Sci Rep. 2021 Feb 25;11:4551. doi: 10.1038/s41598-021-83454-z (PMC7907276; doi:10.1038/s41598-021-83454-z)
Supplement: Supplementary file 1 — Supplementary Information. [file 41598_2021_83454_MOESM1_ESM.pdf]

Supplementary Note 1: Reconstruction

As the sample is rotated each detector pixel collects an intensity $I(\theta) = I_n e^{-k(\theta)}$ at discrete (n) angles through a full rotation of the sample; where I_n is the unattenuated radiation intensity from the source to the detector, k is the attenuation caused by the sample along a detected ray $I(n)$ is the measured intensity, see Fig. 1b. Rays from the sample to the detector approximate straight lines, and so the rays reaching the detector can be represented with line integrals. A projection is then the resulting intensity profile at the detector for a rotation angle, and the integral transform that results in $P_\theta(v)$ is the Radon transform.

The equation of a set of parallel rays from a source passing through the specimen to a point v along the detector is:

$$X\cos(\theta) + Y\sin(\theta) - v = 0 \tag{5}$$

Projecting many such rays through a sample with structure $f(X,Y)$ gives:

$$P_\theta(v) = \int_\infty^\infty \int_\infty^\infty f(X,Y)\delta(x\cos(\theta) + y\sin(\theta) - v)dXdY \tag{6}$$

Where $P_\theta(v)$ is the Radon transform of $f(X,Y)$ which represents the contrast image of a 2D slice of the specimen. The Radon transform of an image produces a sinogram.

An inverse Radon transform is used to recover the original object from the projection data; which is achieved by taking the Fourier transform of each projection measurement, then reordering the information from the sample into the respective position in Fourier space. This is valid due to the Fourier Slice theorem (12) , which states that the Fourier transform of a parallel projection is equivalent to a 2D slice of the Fourier transform of the original sample. The back projection step is given by:

$$f_{\text{fpb}}(X,Y) = \int_0^\pi Q_\theta(X\cos(\theta) + Y\sin(\theta), \theta)dXdY \tag{7}$$

Where Q_θ is the filtered projection data, and $f_{\text{fpb}}(X,Y)$ is the back-projected image (13). A spatial filtering step is applied during back-projection to avoid spatial frequency oversampling during the object's rotation a high pass filter is commonly used to compensate for the perceived blurring. The blurring arises as Q_θ is back-projected (smeared) across the image plane for each angle of reconstruction; which means that not only does the back-projection contribute at the line it is intended to (along line C in Fig. 1b), but all other points along the back-projecting ray.

Now, suppose we know the relative positions of the two cameras and their respective intrinsic parameters, such as magnification and pixel offset. For a single camera and given the camera parameters, we can translate pixel coordinates, $\mathbf{w} = (u,v)$, into the coplanar image plane coordinates $\mathbf{x} = (x,y)$:

$$u = u_0 + k_u x \quad \textbf{(8)}$$

$$v = v_0 + k_v y \quad \textbf{(9)}$$

Knowing the focal length (f) of the imaging system, image plane coordinates may be projected into a ray in 3D. The ray can be defined by using the point $\mathbf{p}$ in camera-centred coordinates, where it crosses the image plane.

$$\mathbf{p} = \begin{bmatrix} x \\ y \end{bmatrix} \quad \textbf{(10)}$$

From the definition of a world point, as observed through an image, we can construct a dual-view model of world points in space as in Fig. 2. Using a model of a system with two views allows for the triangulation of rays based on image correspondences; this is an important part of stereo-vision. The most important matching constraint which can be used is the *epipolar constraint*, and follows directly from the fact that the rays must intersect in 3D space. Epipolar constraints facilitate the search for correspondences, they constrain the search to a 1D line in each image. To derive general epipolar constraints, one should consider the epipolar geometry of two cameras as seen in Fig. 2

The **baseline** is defined as the line joining the optical centres. An **epipole** is the point of intersection of the baseline with the image plane and there are two epipoles per feature, one for each camera. An **epipolar line** is a line of intersection of the epipolar plane with an image plane. It is the image in one camera of the ray from the other camera's optical centre to the world point ($\mathbf{X}$). For different world points, the epipolar plane rotates about the baseline. All epipolar lines intersect the epipole.

The epipolar line constrains the search for correspondence from a region to a line. If a point feature is observed at $\mathbf{x}$ in one image frame, then its location $\mathbf{x}'$ in the other image frame must lie on the epipolar line. We can derive an expression for the epipolar line. The two camera-centered coordinate systems $\mathbf{X}'_\mathbf{c}$ and $\mathbf{X}_\mathbf{c}$ are related by a rotation, $\mathbf{R}$ and translation, $\mathbf{T}$ (see in Fig. 2) as follows:

$$\mathbf{X}'_\mathbf{c} = \mathbf{R}\mathbf{X}'_\mathbf{c} + \mathbf{T} \quad \textbf{(11)}$$

A. The Essential matrix.

Taking the scalar product of Eq. (11) with $\mathbf{X}'_\mathbf{c}$, we obtain:

$$\mathbf{X}'_\mathbf{c} \cdot (\mathbf{T} \times \mathbf{X}_\mathbf{c}) = \mathbf{X}'_\mathbf{c} \cdot (\mathbf{T} \times \mathbf{R}\mathbf{X}'_\mathbf{c}) \quad \textbf{(12)}$$

$$\mathbf{X}'_\mathbf{c} \cdot (\mathbf{T} \times \mathbf{R}\mathbf{X}_\mathbf{c}) = 0 \quad \textbf{(13)}$$

A vector product can be expressed as a matrix multiplication:

$$\mathbf{T} \times \mathbf{X}_\mathbf{c} = \mathbf{T}_\times \mathbf{X}_\mathbf{c} \quad \textbf{(14)}$$

where

$$\mathbf{T}_\times = \begin{bmatrix} 0 & -T_z & T_y \\ T_z & 0 & -T_x \\ -T_y & T_x & 0 \end{bmatrix} \quad \textbf{(15)}$$

So equation Eq. (11) can be rewritten as:

$$\mathbf{X}'_\mathbf{c} \cdot (\mathbf{T}_\times \mathbf{R}\mathbf{X}_\mathbf{c}) = 0 \quad \textbf{(16)}$$

$$\mathbf{X}'_\mathbf{c}\mathbf{T}\mathbf{E}\mathbf{X}_\mathbf{c} = 0 \quad \textbf{(17)}$$

where

$$\mathbf{E} = \mathbf{T}_{\times}\mathbf{R} \tag{18}$$

$\mathbf{E}$ is a 3×3 matrix known as the *essential matrix*. The constraint also holds for rays $\mathbf{p}$, which are parallel to the camera-centered position vectors $\mathbf{X_c}$:

$$\mathbf{p'}^T\mathbf{E}\mathbf{p} = 0 \tag{19}$$

This is the epipolar constraint. If a point $\mathbf{p}$ is observed in one image, then its position $\mathbf{p}'$ in the other image must lie on the line defined by Equation Eq. (19). The essential matrix can convert from pixels on the detector to rays $\mathbf{p}$ in the world, assuming a calibrated camera (intrinsic properties are known), and pixel coordinates can then be converted to image plane coordinates using:

$$\begin{bmatrix} u \\ v \\ 1 \end{bmatrix} = \begin{bmatrix} k_u & 0 & u_0 \\ 0 & k_v & v_0 \\ 0 & 0 & 1 \end{bmatrix} \begin{bmatrix} x \\ y \\ 1 \end{bmatrix} \tag{20}$$

We can modify this to derive a relationship between pixel coordinates and rays:

$$\begin{bmatrix} u \\ v \\ 1 \end{bmatrix} = \begin{bmatrix} \frac{k_u}{f} & 0 & \frac{u_0}{f} \\ 0 & \frac{k_v}{f} & \frac{v_0}{f} \\ 0 & 0 & \frac{1}{f} \end{bmatrix} \begin{bmatrix} x \\ y \\ f \end{bmatrix} \tag{21}$$

$\widetilde{\mathbf{K}}$ is defined as follows:

$$\widetilde{\mathbf{K}} = \begin{bmatrix} fk_u & 0 & u_0 \\ 0 & fk_v & v_0 \\ 0 & 0 & 1 \end{bmatrix} \tag{22}$$

then we can write pixel coordinates in homogenous coordinates:

$$\widetilde{\mathbf{w}} = \widetilde{\mathbf{K}}\mathbf{p} \tag{23}$$

B. The Fundamental matrix.

From Eq. (19) the epipolar constraint becomes

$$\widetilde{\mathbf{w'}}^T\widetilde{\mathbf{K}}^{-T}\mathbf{E}\widetilde{\mathbf{K}}^{-1}\widetilde{\mathbf{w}} = 0 \tag{24}$$

$$\widetilde{\mathbf{w'}}^T F\widetilde{\mathbf{w}} = 0 \tag{25}$$

The (3×3) matrix $\mathbf{F}$, is the called the *fundamental matrix*. With intrinsically calibrated cameras, structure can be recovered by triangulation. First, the two projection matrices are obtained via a SVD of the essential matrix, the SVD of the essential matrix is given by:

$$\mathbf{E} = \mathbf{K'}^T\mathbf{F}\mathbf{K} = \mathbf{T}_{\times}\mathbf{R} = \mathbf{U}\Lambda\mathbf{V}^T \tag{26}$$

It can be shown that

$$\hat{\mathbf{T}_{\times}} = U\begin{bmatrix} 0 & 1 & 0 \\ -1 & 0 & 0 \\ 0 & 0 & 0 \end{bmatrix} U^T \tag{27}$$

and

$$\mathbf{R} = U\begin{bmatrix} 0 & -1 & 0 \\ 1 & 0 & 0 \\ 0 & 0 & 1 \end{bmatrix} V^T \tag{28}$$

Then, aligning the left camera and world coordinate systems gives the projection matrices:

$$\mathbf{P} = \mathbf{K}\left[\mathbf{I} \mid \mathbf{0}\right] \tag{29}$$

and

$$\mathbf{P}' = \mathbf{K}'\left[\mathbf{R} \mid \mathbf{T}\right] \tag{30}$$

Where $\left[\mathbf{I} \mid \mathbf{0}\right]$ is the identity matrix augmented column-wise with a zero matrix, and the two projection matrices ($\mathbf{P}$ and $\mathbf{P}'$) project from camera pixel coordinates to world coordinates. Given these projection matrices, scene structure can be recovered (only up to scale, since only the magnitude of $\mathbf{T}$ ($|\mathbf{T}|$) is unknown) using least squares fitting. Ambiguities in $\mathbf{T}$ and $\mathbf{R}$ are resolved by ensuring that visible points lie in front of the two cameras. As with the essential matrix, the fundamental matrix can be factorised into a skew-symmetric matrix corresponding to translation and a 3×3 non-singular matrix corresponding to rotation.

The second approach is less prone to compound errors but relies on precise identification and tracking of fiducial markers. distinction and tracking fiducials. Instead of calculating $\mathbf{F}$ between neighbouring images, $\mathbf{F}$ is calculated between the current projection and the very first projection. $\mathbf{F}$ is then decomposed and the transformation matrix is inverted and applied to the back projected volume. The reoriented back projected volumes are summed and finally filtered to remove the additional spatial frequencies imparted from rotating the sample.
